# The Impact of Nitroglycerin on the Evaluation of Coronary Stenosis in Coronary-CT: Preliminary Study in 131 Patients

**DOI:** 10.3390/jcm12165296

**Published:** 2023-08-14

**Authors:** Francesca D’Errico, Francesca Ricci, Alessandra Luciano, Francesco Paolo Sbordone, Mario Laudazi, Daniele Mecchia, Maria Volpe, Flavia Briganti, Alessio Di Landro, Saverio Muscoli, Luca Pugliese, Vincenzo De Stasio, Carlo Di Donna, Francesco Romeo, Francesco Garaci, Roberto Floris, Marcello Chiocchi

**Affiliations:** 1 Department of Biomedicine and Prevention, Division of Diagnostic Imaging, University of Rome “Tor Vergata”, Viale Oxford 81, 00133 Rome, Italy; francescaderrico1403@gmail.com (F.D.); alessandraluciano.med@outlook.it (A.L.); sbordonefpaolo@gmail.com (F.P.S.); mariolauda@gmail.com (M.L.); dmecchia13@gmail.com (D.M.); mariavolpe93mv@gmail.com (M.V.); flaviabriganti4@gmail.com (F.B.); l.pugliese88@gmail.com (L.P.); destasio.vincenzo@gmail.com (V.D.S.); didonnacarlo@gmail.com (C.D.D.); garaci@gmail.com (F.G.); roberto.floris@uniroma2.it (R.F.); marcello.chiocchi@gmail.com (M.C.); 2 Unit of Cardiology and Interventional Cardiology, Policlinico Tor Vergata, Viale Oxford 81, 00133 Rome, Italy; aledil01@hotmail.com (A.D.L.); saveriomuscoli@gmail.com (S.M.); 3 Faculty of Medicine, UniCamillus International Medical University, 00131 Rome, Italy; romeocerabino@gmail.com

**Keywords:** nitroglycerin, CAD, coronary CT, plaque pattern, calcific plaque, stenosis

## Abstract

Background: The sublingual administration of short-acting nitroglycerin (NTG) before coronary computed tomography (CCT) improves the visualization of coronary arteries, causing vasodilatation. The aim of this study was to evaluate whether and how nitroglycerin can influence the concordance between radiologists and cardiologists in the evaluation of vessel stenosis measured in CCT by the former and during the following coronarography by the latter. Methods: We conducted a retrospective analysis of 131 patients who underwent CCT for cardiac symptoms in 2022, followed by coronarography performed six months later because of significant stenosis revealed by the CCT. First, the patients were divided into two groups: an NTG group who received sublingual nitroglycerin before CCT and a non-NTG group who did not because of contraindications. Second, 254 stenoses were measured by two radiologists after CCT and by two interventional cardiologists during the next coronarography; moreover, stenoses were classified on the basis of their location and plaque pattern (calcific, mixed and lipidic). Third, the strength of agreement was evaluated between the two radiologists, between the two cardiologists and finally between the radiologists and cardiologists in order to evaluate whether and how the interdisciplinary discrepancy in stenosis evaluation could change with or without the use of nitroglycerin before CCT and in relation to the different plaque pattern. Results: In the NTG group, the use of nitroglycerine reduced the agreement between radiologists and cardiologists in calcific stenosis but did not change the concordance in the case of mixed or lipidic plaques on the same vessels. Conclusions: The use of sublingual nitroglycerin before CCT may lead to a radiological overestimation of calcific stenosis.

## 1. Introduction

When it was introduced in the early 2000s, coronary CT (CCT) rapidly became the preferred imaging technique for coronary artery disease (CAD) because of its lower invasiveness, cost, greater availability, reduced time consumption, greater accuracy, and significant impact on patient management [1]. In particular, the most recent CT scanners allow for accurate assessment of all major plaque features in terms of composition, attenuation and high-risk factors, which are useful in evaluating the so-called ‘plaque load’ [2], with proven prognostic significance. However, despite improvements in spatial and temporal resolution, the visualization of distal segments and secondary vascular branches is still challenging [3,4]. In recent years, the practice of administering short-acting nitroglycerin (NTG) sublingually (two tablets or two puffs, equivalent to 0.8 mg) before CCT scanning has been more frequently adopted in order to induce coronary vasodilatation, improving the visualization of the vascular lumen [5,6]. Several studies have found that one or two sublingual nitroglycerin puffs (0.4–0.8 mg) increase the coronary diameter, the number of assessable coronary segments and the quality of the imaging as well as the diagnostic accuracy of CCT [5]. A possible disadvantage of the pre-CT administration of NTG could be the risk of overestimating the percentage of stenosis, hypothetically due to the lower reactivity of the vessel in the segment affected by atheromatous pathology [7]. However, there is still an insufficient number of studies aiming to determine the influence of NTG on the evaluation of stenosis in CCT. In this scenario, the first aim of this retrospective study consisted of evaluating the effect of nitroglycerin on the measurement of vessel stenosis in CCT compared with the stenosis resulting from the following coronarographic evaluation required because of the severity of stenoses. The final aim was to evaluate whether and how the interdisciplinary discrepancy in stenosis evaluation between radiologists and cardiologists could change with or without NTG before CCT, especially in relation to the different plaque patterns (calcific, lipidic and mixed).

## 2. Materials and Methods

The study population retrospectively included 131 patients (99 males and 32 females) who underwent CCT in 2022 for cardiac symptoms, followed by coronarography performed six months later because of the presence of severe stenosis revealed at the CCT. All patients included in this study provided written informed consent, encompassing their agreement to undergo the examinations and the utilization of their data for medical research. The absence of the angiographic evaluation in the following six months after CCT in the case of low-risk stenosis, the complete occlusion of the coronary arteries, the presence of widespread and advanced vascular disease of coronary arteries and intra-stent stenoses were listed among the exclusion criteria. First, the patients were divided into two groups; the former was represented by 70 patients who underwent CCT receiving the sublingual nitroglycerin before CCT (NTG group), as suggested by recent guidelines, and the latter was represented by 61 patients who underwent CCT without nitroglycerin (non-NTG group) due to contraindications to the use of nitroglycerin (severe arterial hypotension and recent uptake of phosphodiesterase inhibitor) [8]. Nitroglycerine was administrated as a sublingual spray (NATISPRAY 0.30 mg/dose, sublingual spray, two doses) [8] just 2 min before the CCT examination. By contrast, during the coronary angiography evaluation via radial artery approach, nitrates were not administered, whereas in the transfemoral approach, they were administered only after stenosis estimation if there was a need to avoid intraprocedural vasospasm. The stenoses resulting in the NTG and non-NTG groups after CCT were measured as a percentage of vessel caliber reduction by two different radiologists with more than 10 years of experience in cardiac imaging; in addition, the two radiologists also defined the type of plaque, differentiating between calcific, lipidic and mixed pattern. The same stenoses were measured by two different interventional cardiologists during the following coronarography performed because of the severity of the stenoses measured in CCT.

Statistical analysis was then conducted with IBM SPSS v28 software. First, the descriptive statistics were created. Then, the intraclass correlation coefficient (ICC) was used to estimate the strength of the intradisciplinary concordance between the two radiologists and then between the two cardiologists for each vessel (LM: left main, LAD: left anterior descending artery, LCX: circumflex coronary, OM: left marginal branch, RCA: right coronary, PLB: posterolateral, PDA: posterior descending artery, D: diagonal). Subsequently, once the mean value of the stenosis percentages for each method had been calculated, the strength of interdisciplinary concordance between the mean score of the radiologists and that of the cardiologists was assessed, dividing the patients between those who received sublingual nitroglycerin (NTG group) and those who did not (non-NTG group), again using the ICC. Finally, the interdisciplinary concordance between the mean score of the radiologists and that of the cardiologists was also analyzed comparing subgroups of different plaque patterns, only when the size of the sample allowed us to make this final comparison; indeed, this comparison was available only in case of stenosis in LAD, LCx and RCA, which also represents the most common locations of CAD. For all tests, the significance level considered was *p* < 0.05.

## 3. Results

As regards mean age (71 years old), sex, heart rate, body mass index and the dose of beta-blockers administered before CCT, no statistically significant differences were found between the NTG and non-NTG groups before the exam (Table 1). 

**Figure 1:**
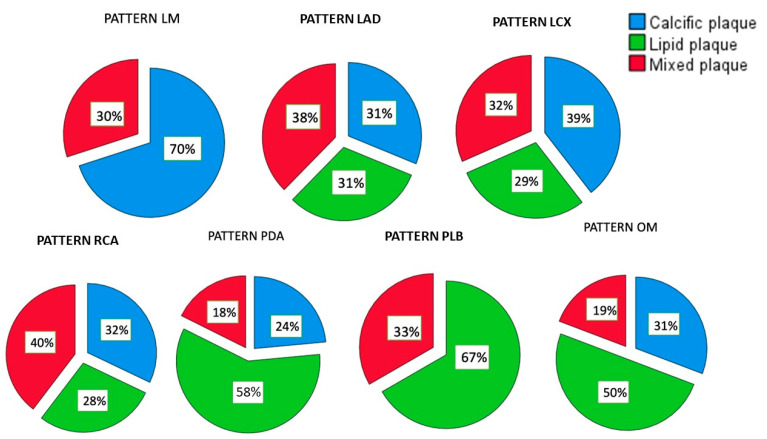
Distribution of plaque pattern type in LM, LAD, RCA, LCx, OM, PDA and PLB. The green part of every diagram represents the subgroup percentages for lipidic plaque, the red one for the mixed plaque and the blue one for the calcific plaque.

The quantitative percentage of different plaque pattern types for each branch is shown in Figure 1.

The analysis of the intraclass correlation coefficient (ICC), to evaluate the strength of agreement between the two radiologists, revealed statistically significant and high/very high concordance (*p*-value < 0.05, ICC between 0.8 and 1) for all coronary vessels examined, except for PLB, whose agreement between radiologists could not reach significance (only three cases). Similarly, agreement between the two cardiologists was statistically significant and high/very high (*p*-value < 0.05, ICC between 0.8 and 1) for all coronary vessels, except for the left main (LM), which showed a significant but moderate concordance between the two cardiologists (ICC of 0.699).

To evaluate the impact of nitroglycerin on the CCT evaluation in the comparison between the NTG and non-NTG groups, this study revealed a significant and low concordance (ICC 0.575 with *p*-value < 0.05) between radiologists and cardiologists in the NTG group for LM. Due to the small sample size, the concordance in the non-NTG group could not be statistically evaluated. Additionally, the study could not assess how the grade of agreement could change between the NTG and non-NTG groups by varying the plaque pattern of LM.

**Figure 2:**
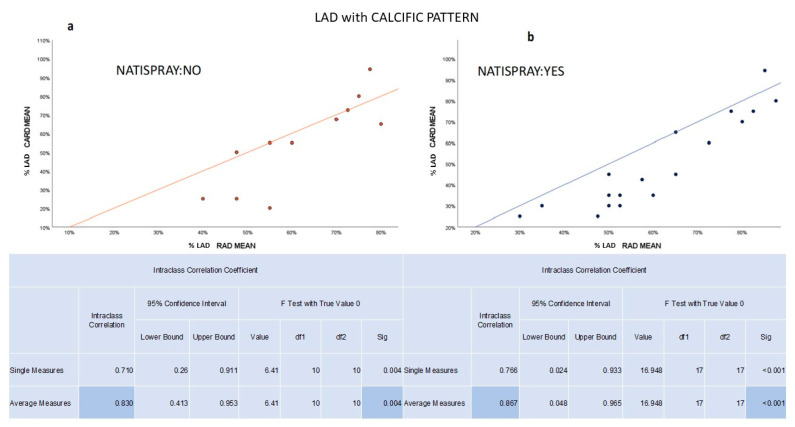
Significant (*p* value <0.05) and high concordance between the mean values of radiologists and the mean values found by cardiologists measuring the stenosis of calcific plaque in LAD in both groups: (**a**) non-NTG and (**b**) NTG.

As for LAD, both the NTG (ICC 0.904) and non-NTG (ICC 0.866) groups exhibited significant (*p*-value < 0.05) and high/very high concordance between radiologists and cardiologists. The strength of the agreement did not change by varying the type of pattern of the plaque (calcific, lipidic and mixed); in particular, it remained high/very high (Figure 2).

**Figure 3:**
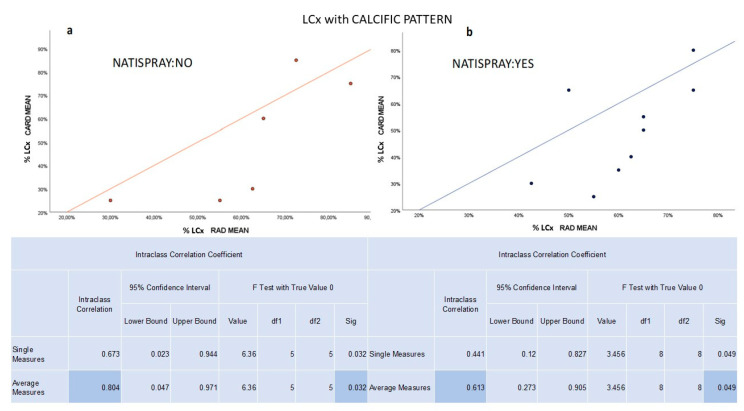
(**a**) In non-NTG group, the graph shows a significant and high concordance between radiologists and cardiologists in evaluating the percentage of stenosis for calcific plaques in LCx. (**b**) In NTG group, the graph shows a significant and medium concordance between radiologists and cardiologists in evaluating the percentage of stenosis for calcific plaques in LCx.

For LCx, we confirmed a significant (*p* value < 0.05) and high/very high concordance between radiologists and cardiologists in both the NTG (ICC 0.808) and non-NTG groups (ICC 0.886). The strength of agreement remained high/very high by varying the pattern of the plaque in the non-NTG group. By contrast, the agreement decreased from high to medium in the NTG group in the case of calcific plaque only due to an overestimation by radiologists (Figure 3).

The analysis of stenosis in OM showed a significant and moderate concordance in the NTG group (ICC 0.774, with *p* value < 0.05) and a very high concordance in the non-NTG group (ICC 0.903, *p* value < 0.05). Due to the small sample size, we could not evaluate how the grade of agreement could change between the NTG and non-NTG groups by varying the plaque pattern.

For both RCA and PDA vessels, significant and high concordance was observed between radiologists and cardiologists in the NTG group (ICC RCA 0.869 and ICC PDA 0.815, respectively) and a significant and very high concordance in the non-NTG group (ICC RCA 0.932 and ICC PDA 0.993, respectively).

**Figure 4:**
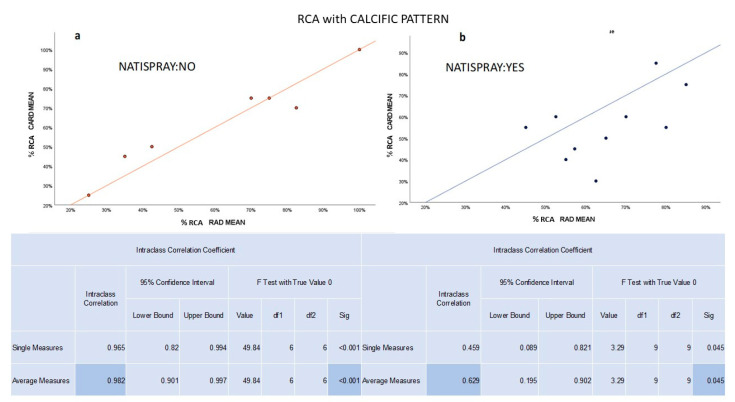
(**a**) In the non-NTG group, the graph shows a significant and very high concordance between radiologists and cardiologists in evaluating the percentage of stenosis for calcific plaques in RCA. (**b**) In the NTG group, the graph shows a significant and medium concordance between radiologists and cardiologists in evaluating the percentage of stenosis for calcific plaques in RCA.

Although the study could not evaluate the agreement change between the NTG and non-NTG groups by varying the plaque pattern in PDA due to insufficient sample size, the strength of agreement remained unchanged for RCA (high/very high), regardless of the plaque pattern in the non-NTG group. In contrast, in the NTG group, the agreement for RCA decreased from high to medium for the calcific plaque pattern only due to an overestimation by radiologists (Figure 4).

From the evaluation of D, the concordance between radiologists and cardiologists in the NTG group was significant and moderate (ICC 0.798 with *p* value < 0.05). However, the agreement in the non-NTG group was not significant. Similarly, the ICC analysis of PLB was not statistically significant in the NTG and non-NTG groups. As mentioned before, for LAD, LCx and RCA, there was concordance between radiologists and cardiologists, as examined, considering the stenoses in relation to the nitroglycerin administration combined with the different plaque patterns (calcific, lipidic or mixed). As a result, from the evaluation of the impact of nitroglycerin (NTG group) on the measurement of the stenosis concerning the different plaque patterns of LAD, LCx and RDA, we concluded that lipidic and mixed patterns did not change the concordance in the NTG group, while the calcific pattern reduced the concordance from high/very high to medium in LCx and RCA in the NTG group, and it did not alter the high agreement in LAD in the NTG group. In the non-NTG group, the result of agreement remained stable (high/very high).

## 4. Discussion

**Figure 5:**
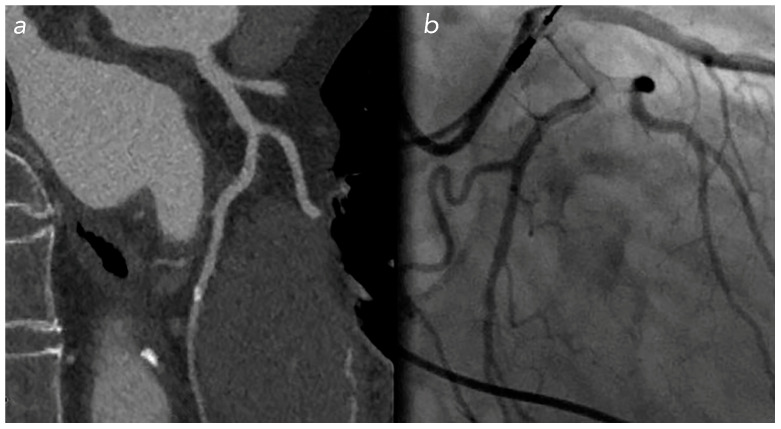
Calcific stenosis on LCx in patient with NTG: overestimation of stenosis percentage in CCT (**a**) compared with the real stenosis found during coronary angiography (**b**).

**Figure 6:**
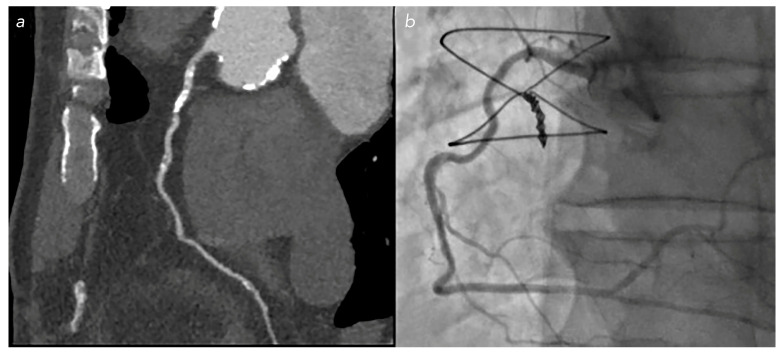
Calcific stenosis on RCA in patient with NTG: overestimation of stenosis percentage in CCT (**a**) compared with the real stenosis found during coronary angiography (**b**).

Currently, the “gold standard” for the diagnosis of CAD is coronary angiography, although in clinical practice, its indications are limited because of the bleeding risk due to its invasiveness, radiation dose and costs [9]. Therefore, in the last decade, CCT has assumed a crucial role in the diagnosis and characterization of CAD, reducing invasive procedures and healthcare costs, becoming a first-line test in the evaluation of patients without known CAD who manifest stable chest pain [8,10]. Sublingual nitroglycerin, used in the treatment of angina pectoris episodes, is commonly used before CCT due to its main pharmacological effects: dilation of epicardial coronary arteries and their collateral vessels; relaxation of vascular smooth muscle and reduction in cardiac preload and afterload; reduction in myocardial oxygen consumption and improvement in blood perfusion in the ischemic area [9,11]. Its use in CCT was explored by many authors who demonstrated its ability to improve the quality of the imaging and the diagnostic accuracy, increasing the coronary diameter and the number of evaluable segments [5,8,12,13] with limited side effects [14]. Indeed, it has been reported that nitroglycerin increases the luminographic area of the vessel lumen by 20% to 40% in healthy arteries and between 5% and 10% in arteries with disease [15], improving the evaluation of major branches and of secondary segments [16]. However, few studies have evaluated the overestimation risk of coronary stenosis in CCT due to nitroglycerin-induced vasodilation [17]. Conti et al. demonstrated that in 119 coronary artery stenoses, evaluated using conventional angiography, the use of sublingual nitroglycerin resulted in a significant increase, around 8%, in the percentage of stenosis [7,18]. The main aim of our study was to evaluate whether there was any overestimation of the percentage of coronary stenosis related to the administration of sublingual nitroderivate in CCT in regard to the different features of the plaque (calcific, mixed or lipidic). If we do not consider the type of plaque pattern, the results showed that the use of nitroglycerin did not affect the concordance between radiologists (except for PLB, probably due to the small sample size) nor the interdisciplinary concordance between radiologists and cardiologists on the evaluation of the stenosis of the main coronary branches, except for OM, where the interdisciplinary concordance was moderate because of an overestimation of stenosis by radiologists in the NTG group. Therefore, the most interesting result emerged from the subanalysis, which related the interdisciplinary agreement with the use of NTG combined with the plaque pattern available only for LAD, RCA and LCx. Indeed, we found that the use of NTG in the evaluation of the calcific pattern caused an overestimation of the percentage of stenosis by radiologists in CCT (Figure 5 and Figure 6).

As regards lipidic and mixed plaque patterns, the use of sublingual NTG did not cause a significant alteration in the interdisciplinary agreement between radiologists and cardiologists in the evaluation of stenosis. The explanation of these results is inside the mechanism of action of nitroderivatives on the vessel endothelium before the calcific metaplasia occurs. Indeed, in CAD, exogenous organic nitrates like nitroglycerin are converted to nitric oxide (NO), normally produced by a healthy endothelium, which promotes smooth muscle relaxation, vasodilation, reduces vasospasm and prevents platelet aggregation. The decrease in vasodilation capacity in atherosclerosis is mainly related to the decreased endothelium-derived NO. Exogenous nitrates, on the other hand, act with the same mechanism but are endothelium-independent vasodilators. For that reason, they exert their relaxing action, allowing for vasodilation of the vessel smooth muscle, even if mixed or lipidic endothelial plaques are present [19]. However, in calcific atheroma, the progression of calcification occurs from the outer edge of the necrotic core to the collagen matrix, involving the smooth muscle cells of the middle tonaca [20]. This phenomenon could cause the vessel wall to become less sensitive to the effects of nitric oxide, making the vessel stiffer, especially in the case of concentric plaques [7]. For these reasons, the administration of nitroglycerin in a coronary artery with calcific atheromasic pathology will promote pre-stenosis and post-stenosis vasodilation, but it will have a trivial effect on the calcific stenotic tract, thus resulting in an overestimation of stenosis in CCT [13]. These data could explain the overestimation of stenosis by radiologists in the NTG group combined with the calcific pattern of the plaque. However, this study has some limitations. First, the sample is small and from a single center; therefore, the statistical analysis of the interdisciplinary agreement for the plaque pattern subgroups was available only for LAD, LCx and RCA. The characteristics of the study population, with a higher proportion of males (75.57%) and an older age (mean age of approximately 71 years), may limit the observed results to gender- and age-related patients. Lastly, because of the retrospective nature of the study, it was not possible to evaluate the interesting effect of nitrate on heart rate and blood pressure of the patient after the sublingual administration.

## 5. Conclusions

In conclusion, our study found that NTG had minimal impact on the overall agreement between radiologists and cardiologists in coronary stenosis evaluation using CCT. However, NTG administration with calcific plaques overestimated stenosis in certain coronary vessels (LCx and RCA), while lipidic and mixed plaques showed minimal impact. Considering the plaque composition during NTG-assisted CCT evaluation is crucial [21]. As mentioned before, the advantage in terms of diagnostic accuracy of using sublingual nitroglycerin in CCT is approved by several authors. Our work confirms this advantage, but it also mentions the possibility of an overestimation of stenosis after NTG administration that radiologists should consider when they evaluate calcific plaques.

## Figures and Tables

**Table 1:** Population baseline characteristics of the patients, with descriptive statistics both global and divided by subgroups.

Patient Characteristics	NTG Group	Non-NTG Group	Global Population
Nr	70	61	131
Female (%)	18	14	32 (24.4%)
Male (%)	52	47	99 (75.6%)
Age Mean ± SD	71.05 ± 9.21	71.45 ± 9.73	71.24 ± 9.42
Age Median	72.5	73	73
Age Min	48	45	45
Age Max	90	87	90
BMI Avg	28.03	27.71	27.80
HR Avg	70	68	67

## Data Availability

All data generated and analyzed during this study are included in this published article. The datasets used and analyzed are also available from the corresponding author on reasonable request.
